# Criticisms of the nutrient-dependent pheromone-controlled evolutionary model

**DOI:** 10.3402/snp.v4.24367

**Published:** 2014-06-04

**Authors:** Andrew Jones

Dear Dr. Mouras,

Despite his valid publications involving endocrinology, sexuality, and epigenetically induced intraspecies differentiation in model organisms, James V. Kohl overextends his expertise in trying to overthrow established evolutionary theory. His earlier publications cover topics such as behavioral effects of hormones, pheromones, and food odors (Kohl, 1996, 2012). However, in 2013, he submitted a manuscript to Socioaffective Neuroscience & Psychology in which he attempted to link his previous work on behavior and its development to larger, overarching, evolutionary concepts. This Letter to the Editor is a criticism of both his published works and external discussions in which he attempts to clarify his position (Kohl, 2013a, b).

In this latest paper, Kohl posits that evolution is *exclusively* driven by genetically predisposed, nutrient-dependent, and pheromone-controlled behavior and sexual selection. On the molecular level, he references only epigenetic processes (although he does not describe them in detail) which affect when genes are transcribed, reversible alterations like genome methylation, silencing, control of splicing, chromatin remodeling, and so on. There is no mention at all of any biochemical pathways or enzymes that are involved in ‘nutrient-dependent, pheromone-controlled’ nucleotide or amino acid substitutions, so how changes in the DNA sequence are made in his model are entirely unexplained. However, Kohl heavily implies, without directly stating, that alternative splicing (the only mechanism he does specifically mention) is responsible for genetic diversity, which is false because splicing does not have the capability to make changes to the genome itself. He explicitly denies the contribution of mutations to genotypic and phenotypic variety, claiming that ‘biophysical constraints’ prevent ‘constraint-breaking mutations’ (phrases taken from Nei, 2013) from being involved in evolution entirely (Kohl, 2014a). Mutations are, according to him, only involved in disease and cannot result in adaptive traits, despite the massive amount of evidence contrary to that.

In addition, Kohl demonstrates a blatant disregard for established nomenclature. For example, he routinely attempts to redefine ‘natural selection’. Charles Darwin (1859) defined it on page 61 of *On the Origin of Species* as the ‘principle, by which each slight variation, if useful, is preserved’. He often defines it as selection *by* the organism *for* nutrients (Kohl, 2013a) instead of the typical selection *by* the environment (biotic or abiotic) *for* advantageous phenotypes. Another term coined by Darwin, ‘conditions of life’, is frequently described by Kohl as ‘nutrient-dependent and pheromone-controlled’ (Kohl, 2013b). Seeing that the conditions of life are the external circumstances to which an organism must be adapted, the descriptors ‘nutrient-dependent and pheromone-controlled’ are not at all applicable to the environment.

Kohl also shows significant comprehension issues within his own paper and in external discussions of references he believes support his model. In the ‘Insects’ subsection of ‘An epigenetic continuum …’ within his 2013 paper (Kohl, 2013a), he briefly mentions the well-known peppered moth example of evolution by selective predation. He denies that predation was the driving force, followed by a seemingly irrelevant statement and citation concerning the moths’ migration. In an external discussion, Kohl has attributed the melanism to a change in the moths’ diet brought on by the pollution, despite the fact that this hypothesis has been contradicted by experimental and statistical evidence stemming from three separate studies (Prakash, 2006). In the ‘Mammals’ subsection, he begins by stating that mutations theory does not address pleiotropy or epistasis. The citation he uses here says nothing about either of those processes or their relation to mutations, so it does not support his assertion. Kohl then refers to an allele change that occurred in a population in China 30,000 years ago as ‘probably … nutrient-dependent’ without making reference to what nutrient caused the change or how. This is followed by the statement ‘the effect … is due to an epigenetic effect of nutrients on hormones responsible for the tweaking of immense gene networks’. Allele changes are not epigenetic and I know of no mechanism that makes deterministic gene sequence changes prompted by epigenetic alterations. A multitude of misconceptions and misunderstandings can be seen in his comments on Dr. PZ Myers’ blog, *Pharyngula* (Kohl, 2014b). For example, in comment #125, Kohl says that proteasomes mediate protein folding. Proteasomes do no such thing. They are actually structures whose function is to break down proteins. In that same post, he reiterates his lack of knowledge of natural selection by asking another commenter to ‘indicate how a beneficial mutation somehow knew it would be beneficial’.

Based on his writings, both published and unpublished, James Kohl presents an unsupported challenge to modern evolutionary theory and misrepresentations of established scientific terms and others’ research. It was a mistake to let such a sloppy review through to be published.

Andrew Jones, BA

ajones4@carthage.edu

**Editor's note**

The 2013 review article by James Vaughn Kohl published in Socioaffective Neuroscience & Psychology and criticized in the above *Letter to the Editor* was subjected to standard peer review and the revised version was accepted by me after it had been accepted by both reviewers.

*Harold Mouras*
Editor-in-Chief
